# An Insight in the Reproductive Biology of *Therophilus javanus* (Hymenoptera, Braconidae, and Agathidinae), a Potential Biological Control Agent against the Legume Pod Borer (Lepidoptera, Crambidae)

**DOI:** 10.1155/2017/3156534

**Published:** 2017-09-28

**Authors:** Djibril Aboubakar Souna, Aimé Bokonon-Ganta, Marc Ravallec, Antonino Cusumano, Barry Robert Pittendrigh, Anne-Nathalie Volkoff, Manuele Tamò

**Affiliations:** 1 UMR DGIMI 1333 INRA, UM, Case Courrier 101, Place Eugène Bataillon, 34 095 Montpellier, France; 2 International Institute of Tropical Agriculture, Benin Research Station (IITA-Benin), 08 BP 0932 Cotonou, Benin; 3 Faculté des Sciences Agronomiques (FSA), Université d’Abomey Calavi, 01 BP 526 Cotonou, Benin; 4 Department of Plant Sciences, Wageningen University & Research, Wageningen, Netherlands; 5 Department of Entomology, Michigan State University (MSU), East Lansing, MI, USA

## Abstract

*Therophilus javanus* is a koinobiont, solitary larval endoparasitoid currently being considered as a biological control agent against the pod borer Maruca vitrata, a devastating cowpea pest causing 20–80% crop losses in West Africa. We investigated ovary morphology and anatomy, oogenesis, potential fecundity, and egg load in *T. javanus*, as well as the effect of factors such as age of the female and parasitoid/host size at oviposition on egg load. The number of ovarioles was found to be variable and significantly influenced by the age/size of the *M. vitrata* caterpillar when parasitized. Egg load also was strongly influenced by both the instar of *M. vitrata* caterpillar at the moment of parasitism and wasp age. The practical implications of these findings for improving mass rearing of the parasitoid toward successful biological control of *M. vitrata* are discussed.

## 1 Introduction

The legume pod borer Maruca vitrata (Fabricius) (syn. *M. testulalis*) (Lepidoptera: Crambidae) is an important pest of cowpea, Vigna unguiculata L. Walp (Fabales: Fabaceae), a widely cultivated legume crop in Sub-Saharan Africa, and can cause yield losses in the range of 20–80% [1]. According to taxonomic and population genetic studies, the putative area of origin of this pest is assigned to South Asia [2]. *Therophilus javanus* (Bhat & Gupta, 1977) (Hymenoptera: Braconidae) is an endoparasitoid that develops inside *M. vitrata* during the early larval stages. High parasitism rates of *T. javanus* on *M. vitrata* caterpillars have been reported in soybean and yard-long beans fields in tropical Asia, Lao PDR, Vietnam, and Southern Taiwan [3, 4]. *T. javanus* thus seems an excellent candidate for use as a biological control agent against *M. vitrata* in West Africa. However, the development of biological control relying on *T. javanus* releases requires a thorough knowledge of its basic biology, which has not been investigated yet.

*Therophilus javanus* belongs to the Agathidinae subfamily of Braconidae, which includes an estimated two to three thousand species worldwide with a higher number of genera in tropical than in temperate regions [5]. Some species have been employed as biological control agents against various insect pests [6, 7]. Although Agathidinae have been studied for taxonomic or phylogeny purposes, the biology of members of this subfamily remains largely unknown. A few biological and quite ancient studies have been conducted on Agathidinae oviposition and larval development [8–11]. Most studied Agathidinae species oviposit into special organs (nerve ganglia) [12], but some, including *T. javanus*, place their eggs directly into the host hemocoel [8–10]. Apart from a few studies, that is, the number of eggs laid by Bracon vulgaris (Hymenoptera: Braconidae) [11], and the number of offspring produced by *Agathis gibbosa* (Hymenoptera: Braconidae) [9], there is a dearth of data concerning Agathidinae reproductive biology.

Fecundity is one of the proxies used by biologists to investigate the individual fitness [13] and may greatly vary depending on the species and its life cycle. It can also be affected by a series of abiotic (e.g., temperature) and biotic (e.g., wasp nutritional status, mating status, and age) parameters. Fecundity has been shown to correlate positively with the number of ovarioles, that is, the egg-producing components of the ovary [14]. The number of ovarioles is commonly species-specific, and there is great variation across insects, ranging from less than five per ovary in some flies to hundreds per ovary in some grasshoppers [15]. As such, ovariole number is relatively stable for a given species but can vary due to different environmental or nutritional conditions [16]. In some parasitoid species, adults emerge with their full load of mature eggs (termed “proovigenic”) while other species mature eggs during their adult life (termed “synovigenic”) [17]. For the latter, production of the first eggs relates to the amount of nutritional resources stored during larval stages [18, 19].

In the present work, we investigated *T. javanus* reproductive biology, ovariole number, egg development, and the potential fecundity, as well as how egg loads vary depending on the wasp female age and how they are affected by parameters such as the nutritional quality provided by the lepidopteran host (i.e., caterpillar instar).

## 2 Materials and Methods

### 2.1 Insect Rearing

*Therophilus javanus* was provided by The World Vegetable Center (AVRDC), Taiwan, Republic of China, and reared for several generations under confined conditions at IITA, Benin research station. The pod borer *M. vitrata* colony was established from feral populations collected from both cowpea fields and alternative host plants surrounding the IITA-Benin station. Insect colonies were reared under laboratory conditions, with 12: 12 L: D photoperiod 26°C ± 1.1°C average temperature and 76% ± 7% relative humidity. Four-day-old, mated adult females of *M. vitrata* were transferred in groups of four or five individuals to transparent cylindrical plastic cups (3 cm diameter × 3.5 cm height) and kept for 24 h to allow for oviposition. Ovipositing females were fed using small pieces of filter paper moistened with 10% honey solution. Cups carrying eggs were kept at the same experimental conditions until hatching by the first instar caterpillars, which were subsequently transferred to large cylindrical plastic containers (11 cm height × 16.5 cm diameter) containing sprouting cowpea grains as a feeding substrate.

Colonies of *T. javanus* were reared on *M. vitrata* first instar (three-day-old) caterpillars submitted to parasitization by *T. javanus* mated females. Parasitized caterpillars were reared on sprouting cowpea grains until pupae stage. Emerged adults were fed with a honey solution.

### 2.2 Reproductive Tract Morphology and Ovary Anatomy

Three-day-old adult females were dissected in a phosphate-buffered saline (PBS) solution to carefully recover the reproductive system. The specimens were prefixed in 2.5% glutaraldehyde in cacodylate buffer at 4°C duringthenight. Once fixed, the samples were washed (3 × 10 min) in cacodylate buffer. Postfixation was performed in 2% osmium tetroxide in the same buffer for 1 h at room temperature. Afterwards, the reproductive systems were carefully rinsed with distilled water and washed (3 × 10 min) in 50%, 70%, 90%, and 100% alcohol. Samples were subsequently placed for 1 h in a solution of EMbed 812 Resin (EMS) diluted at 50% in absolute alcohol, were rested overnight at room temperature, and were then transferred to a second, freshly prepared EMbed 812 Resin for 24 h at +60° C for polymerization. Semithin sections were then obtained using an ultra-microtome and stained with methylene blue.

We also examine the egg development within ovariole by dissecting female wasps in PBS at different time intervals after adult emergence (24, 48, 72, and 96 hours). After dissecting the ovaries, ovarioles were removed and fixed in 4% paraformaldehyde. Samples were washed for 5 minutes in PBS and stained either with DAPI (for DNA staining) or phalloidin (for actin staining) by incubation of the specimens for 30 minutes in a solution containing fluorescent phalloidin and DAPI markers diluted at 1/1000 in PBT1%, respectively. Samples were rinsed for 10 minutes in PBS and distilled water and then dried and stored at +4°C for observation of change in the contents of the ovariole using a fluorescence microscope (Zeiss Axiovert 200M equipped with Zeiss AxioCam MRm). Images were processed with ImageJ software [2020].

### 2.3 Ovariole Counts

To examine the effect of the *M. vitrata* host quality on ovariole number in adult female, one hundred and sixty (160) each of first instar (two-day-old), old first instar (three-day-old), and second instar (four-day-old) *M. vitrata* caterpillars, respectively, were submitted individually to parasitization by three-day-old *T. javanus* females. Caterpillars chosen were well-fed and of uniform size. Each was permitted to be stung once and then reared individually on sprouting cowpea grains in plastic cups (diameter: 9 cm on base and 12 cm on top; height: 4.5 cm) until egression of the parasitoid larva from the host and spinning of the cocoon for the pupal stage. Pupae were then collected in the plastic cups until adult emergence. Thirty-one (31) females (per host group) more than 24 h age were dissected in PBS under a stereomicroscope and the number of ovarioles per ovary was counted.

**Figure 1:**
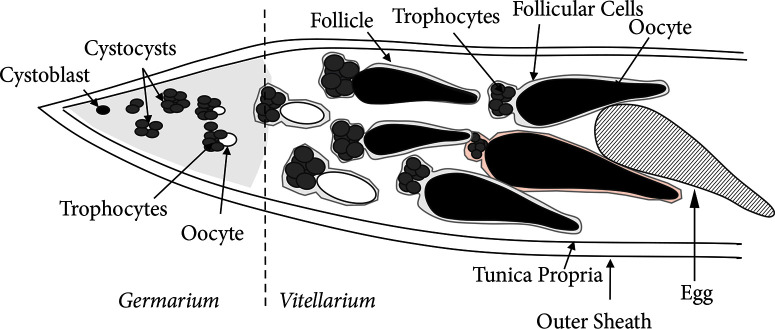
Schematic representation of the ovariole of *Therophilus javanus* showing differentiated oocyte and accompanying nurse cells (trophocytes) within the ovariole. Immature eggs recorded in our enumeration are large individual egg chambers (follicle) located in the vitellarium whose oocyte displayed an ovoid form and had a slender tapering stalk at their posterior end in solid black color, and mature eggs recorded are large individual egg chambers (egg) of ovoid form that had a slender tapering stalk at their posterior end in black striped color.

2.4. Estimation of Egg Production in Female *T. javanus*. Parasitoid females used in this study were mated and fed using 10% honey solution but not allowed to oviposit. Twenty (20) 12-hour-old and twelve (12) 72-hour-old females were dissected in PBS and observed under a stereomicroscope. Because eggs chambers in *T. javanus* are translucent white, dissected ovaries were placed in red neutral solution for five (5) minutes to easily observe immature and mature eggs that were thus colored in red. Each ovariole was then detached and opened in PBS solution to count the number of eggs per ovariole. Large size individual eggs, still accompanied by nurse cells, were categorized as “immature eggs” (indicated by solid black color in Figure 1) and well-formed eggs that displayed an ovoid form and had a slender tapering stalk at their posterior end as “mature” eggs (black striped in Figure 1). We counted both immature eggs and mature eggs to estimate the egg production in *T. javanus*. Small size eggs chambers that were not individually differentiated were not counted (solid white color in Figure 1).

2.5. The Effect of Host Age at Oviposition and Female *T. javanus* Age after Emergence on Mature Egg Production. Maruca vitrata first instar (two-day-old) and second instar (four-day-old) caterpillars, well-fed and of uniform size, were submitted individually for parasitization by three-day-old *T. javanus*. Each was permitted to be stung once and then reared individually on sprouting cowpea grains in plastic cups (diameter: 9 cm on base and 12 cm on top; height: 4.5 cm) until egression of the parasitoid larva from the host and spinning of the cocoon for the pupal stage. Pupae were then collected in the plastic cups until adult emergence. Thirty (30) emerged female wasps of increasing age (one, two, three, four, and five days after adult emergence) were dissected in PBS under a stereomicroscope. Ovaries were removed and placed in red neutral solution for five minutes. The total number of mature eggs (black striped in Figure 1) in the ovariole was counted per ovary.

2.6. Data Analysis. Data were collected from February 2015 to February 2017 and general linear models with Poisson errors and log-link function, corrected for overdispersion, were used, (1) to test which host caterpillar stages impact the ovariole number in female parasitoid; (2) to test the effect of the female age on (i) the number of eggs (immature eggs + mature eggs) per ovariole, (ii) the number of eggs per ovary, and (iii) the number of eggs per female; (3) to probe the link between the number of ovarioles and the number of eggs in females; and (4) to investigate to what extent host caterpillar stage or parasitoid female age impacts the number of mature eggs per female in *T. javanus*. Multiple comparisons were carried out using the glht function of the “multcomp” package in the R software [20] to determine significant differences among the mean number of ovarioles per female (at the 0.05 significance level). The statistical software package R 3.3.2 [21] was used for all statistical analyses.

## 3 Results

### 3.1 General Morphology of *Therophilus javanus* Female Reproductive System

**Figure 2:**
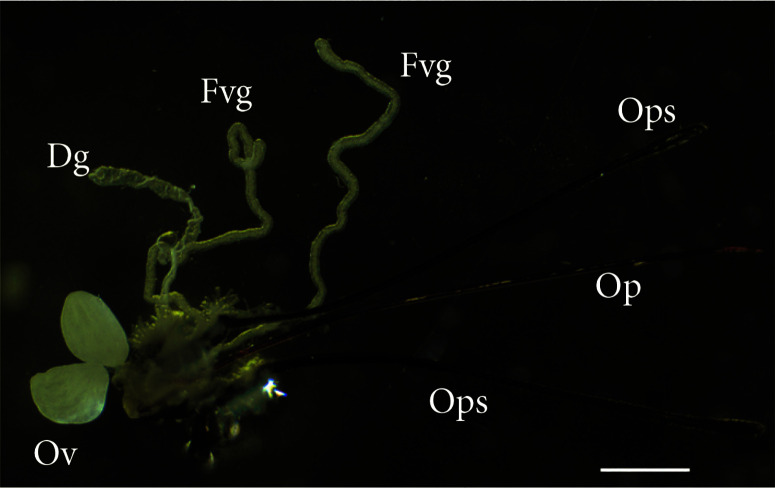
General morphology of *Therophilus javanus* female reproductive system, showing the two ovaries (Ov), the venom gland composed by two filaments (Fvg), Dufour’s gland (Dg), the ovipositor (Op), and the two ovipositor sheaths (Ops). Bar 1 mm.

The *T. javanus* female reproductive system consisted of a pair of globular-shaped ovaries housing several ovarioles, a spermatheca, a Dufour’s gland, a venom gland, composed of a venom duct and two venom gland filaments, and the wasp ovipositor (Figure 2).

### 3.2 Impact of Host Quality on the Number of Ovarioles per Female

**Figure 3:**
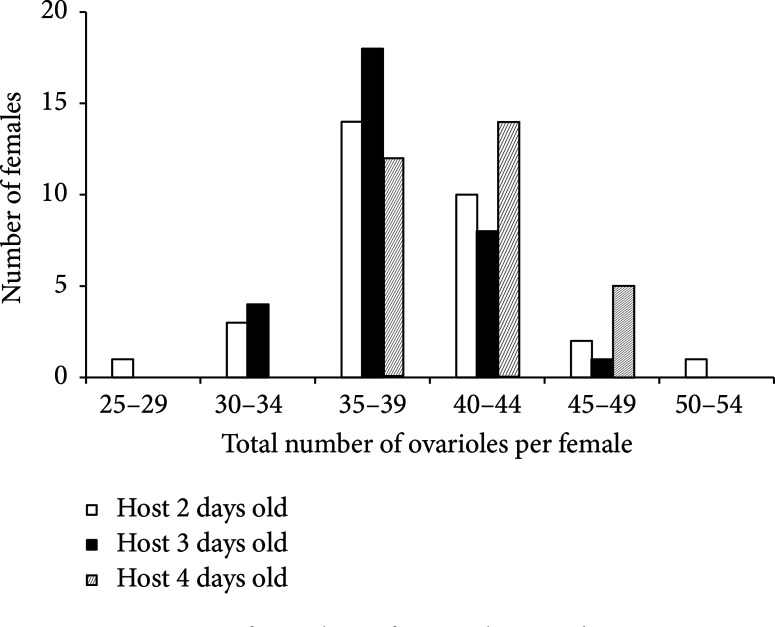
Variation of number of ovarioles in *Therophilus javanus* female (*n* = 31) depending on the host caterpillar age at time of parasitism (two-day-old caterpillars, three-day-old caterpillars, and four-day-old caterpillars).

The mean number of ovarioles per female was found to be significantly influenced by host age at the moment of oviposition (GLM: χ2 = 3.6358, df = 2, p < 0.05) (Figure 3). In general, the number of ovarioles varied between the three females categories and was increased as the host caterpillar increased in size at the moment of oviposition. The average count in one-day-old females emerging from L1 two days old, L1 three days old, and L2 four days old is 38.36 ± 4.42 (n = 31); 38.16 ± 3.20 (n = 31); and 40.87 ± 3.15 (n = 31), respectively.

**Figure 4:**
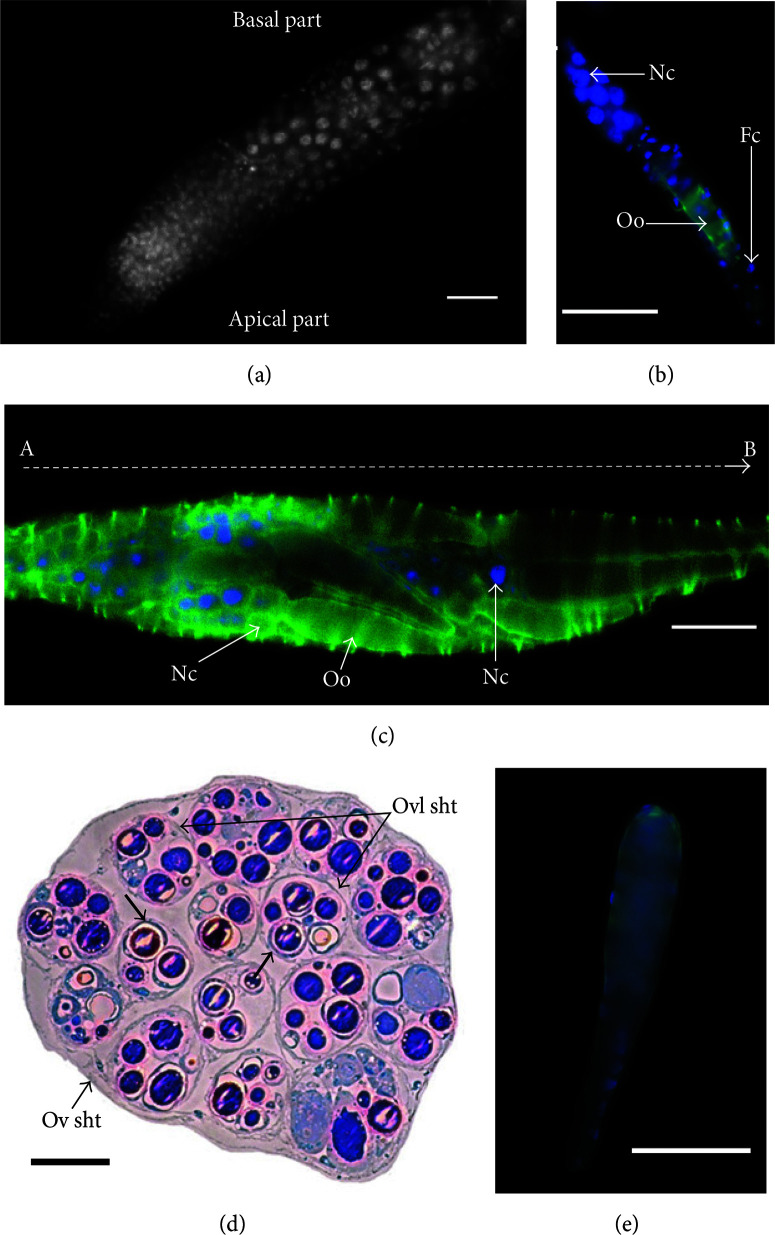
Oogenesis and ovarioles organization in *Therophilus javanus*. (a) View of the anterior region of *T. javanus* ovariole (germarium), indicating cell nuclei that increase in size along the germarium. Bar 20 𝜇m. (b) Individual follicle taken out from a *T. javanus* ovariole. The picture shows the disposition of the nurse cells (Nc) at the top of the oocyte (Oo).The nuclei of follicular cells (Fc) fromthe sheath surrounding the follicle can also be observed. Bar 50 𝜇m. (c) Basal part of the ovariole of *T. javanus*. Follicles are in increasing development stages along the vitellarium (from A to B). On the left of the picture, follicles display small oocytes and trophocytes with a large nucleus. On the right, oocytes have increased in size thanks to progression of vitellogenesis.Note the network of actin fibers (in green) surrounding the egg chamber. Nurse cells (Nc); oocyte (Oo). Bar 50 𝜇m. (d) Organization of the ovarioles and follicles within *T. javanus* ovaries: cross-sections of an ovary containing 15 ovarioles. The ovary is enveloped by the ovarian epithelial sheath (Ov sht) and each ovariole is surrounded by an epithelial sheath (Ovl sht). In the section, oocytes are in different stages within and between ovarioles. Some eggs with a chorion can be observed (shown by arrows). Semithin section stained with methylene blue. Bar 50 𝜇m. (e)The mature egg of *T. javanus*.The egg has an ovoid shape and a slender tapering stalk at its posterior end. Bar 50 𝜇m.

### 3.3 Egg Development within the Ovariole

*Therophilus javanus* ovarioles belong to the polytrophic meroistic type. Egg development occurred anteriorly to posteriorly along the ovariole, with two distinctly recognizable regions: the germarium and the vitellarium. The germarium contained a number of spherical cells observed as either free or clustered (Figure 4(a)). Cell nuclei size increased as they progressed along the germarium. The vitellarium is the posterior region of the ovariole, where egg chambers (follicles) are formed and grown. In *T. javanus* vitellarium, nurse cells were disposed at the top of the oocyte, all being surrounded by a sheath composed of follicular cells (Figure 4(b)). During progression of the follicles from the anterior to the posterior part of the vitellarium, vitellogenesis takes place and the size of the oocyte increases (Figure 4(c)). A cross-section of the entire ovary shows that egg chambers were in different maturation stages within and between ovarioles (Figure 4(d)). Well-differentiated oocytes (with chorion) displayed an ovoid shaped form and had a slender tapering stalk at their posterior end. Mature eggs measured 160.9 ± 6.9 µm (n = 20) in length with widths ranging from 25.3 ± 2.6 µm (n = 20) (anterior pole) to 9.4 ± 1.3 µm (n = 20) (posterior/basal pole) (Figure 4(e)).

### 3.4 Impact of *T. javanus* Female Age on the Number of Eggs

The number of eggs (both immature and mature) per female ranged from 1 to 88 and from 349 to 476 in 12-hour-old and 72-hour-old females, respectively. The overall mean number per female increased with the female age (GLM: χ 2 = 6481.2, df =1, p < 0.001). The number of eggs ranged from 0 to 6 and from 3 to 21 per ovariole, 12 hours and 72 hours after female emergence, respectively (Table 1). As expected, the number of eggs per female was found to be significantly influenced by the total number of ovariole per female (GLM: χ 2 =233.4, df = 1, p< 0.001).

**Table 1:** Egg (immature + mature eggs) number (mean number ± SD) in female*Therophilus javanus* after emergence.Caterpillarswere three days old at the moment of oviposition.

	Number of eggs	
	12 h after emergence	72 h after emergence
Ovariole	0.9 ± 1.2^b^	11.2 ± 2.7^a^
Female	30.7 ± 31.2^b^	407.7 ± 45.7^a^

Mean number of eggs (immature eggs + mature eggs) per ovariole and per female at 12 and 72 h after adult emergence. Eggs that were attached by nurse cells were recorded as immature eggs (black solid color in Figure 1), and well-formed eggs who displayed an ovoid form and had a slender tapering stalk at their posterior end were recorded as mature eggs (black striped color in Figure 1). Means followed by different letters between columns are significantly different between females at 12 h (𝑛 = 20) and 72 h (𝑛 = 12) after adult emergence only according to GLM with “quasi-Poisson distribution” and log-link function (𝑝 < 0.05).

### 3.5 Impact of Host and Female Wasp Age on the Egg Load

**Figure 5:**
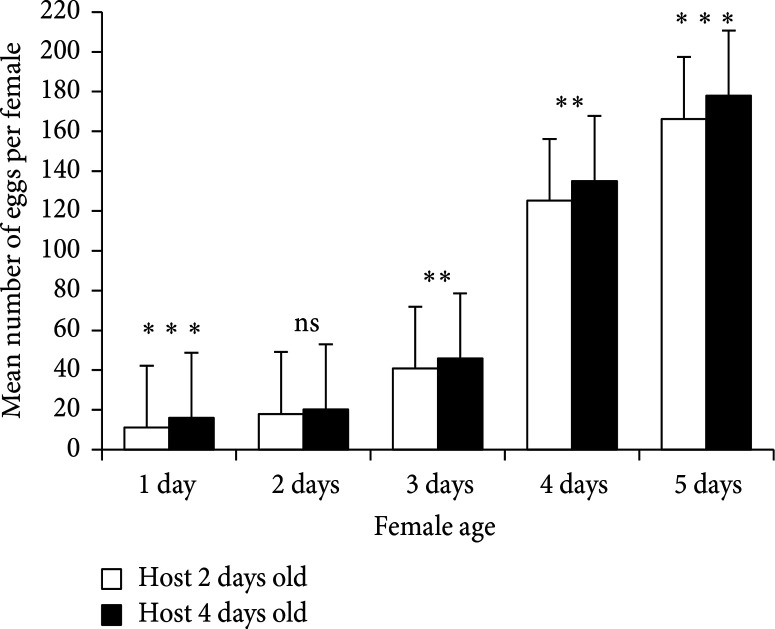
Effect of host age (at oviposition) and female age on the mature egg (individual egg chambers located in the vitellarium who displayed an ovoid form and had a slender tapering stalk at their posterior end (in black striped color in Figure 1)) load in *Therophilus javanus*. Caterpillars were two and four days old at the moment of oviposition. Error bars represent the standard errors of the means (𝑛 = 30). Means that were significantly different between two-and four-day-old hosts only according to GLM and Tukey HSD test are indicated by asterisks (∗∗𝑝 < 0.01; ∗∗∗𝑝 < 0.001; ns: nonsignificant).

Overall, the mean number of mature eggs per female was found to be significantly influenced by host age (GLM: χ2 = 44.4, df = 1, p < 0.001) and parasitoid female age (GLM: χ 2 = 16600.9, df=4, p < 0.001). Females that emerged from four-day-old host caterpillar at oviposition had a higher mean number of mature eggs. (Figure 5).

## 4 Discussion

*Therophilus javanus* belongs to an important braconid family, the Agathidinae, whose reproductive biology is largely unknown. Our study is the first of its kind highlighting major characteristics of *T. javanus* reproductive biology and provides the basis for deploying this parasitoid as a biological control agent against the cowpea pod borer *M. vitrata* in West Africa and elsewhere.

The female reproductive tract of *T. javanus* presents a classical basic morphological organization, similar to the one described in braconids and other Agathidinae species, e.g., Agathis pumila (Hymenoptera: Braconidae) (Ratzeburg) [10]. However, differently from other braconids, the follicles are not organized in a string within *T. javanus* ovarioles but rather appear as “free” egg chambers. This kind of organization resembles to some extent what have been described in some Eulophidae parasitoids (e.g., Palmistichus elaeisis (Hymenoptera: Eulophidae)) [22].

In contrast to the insect model Drosophila, scarce data is available on oocyte size and number and on ovariole number in parasitoid species [23]. In some parasitoid species (e.g., ichneumonids), the number of ovarioles was shown to be a good indicator of fecundity [24]. Ovariole number is largely species-dependent but may show plasticity as a function of biological or environmental factors [25–27]. Our observations suggest that *T. javanus* displays quite a variable number of ovarioles, which has also been commonly reported in noncyclostome Braconidae subfamilies, including in Agathidinae (from 4 to 30) [6, 28]. In *T. javanus*, the highest number of ovarioles has been observed in females issued from larger hosts (i.e., second instar *M. vitrata* larvae). Impact of host instar on parasitoids ecological and biological traits has been reported in several studies [29–32]. For example, the average number of eggs in Microplitis rufiventris Kok (Hymenoptera: Braconidae) was higher in females that emerged from Spodoptera littoralis (Boisd.) (Lepidoptera: Noctuidae) parasitized at younger larval stage [33].

Our study has demonstrated that, in *T. javanus*, egg load is influenced by females’ age. The largest average number of mature eggs (177.97 ± 2.62) was counted in five-day-old females that emerged from larger hosts (L2, four-day-old). This confirms observations in other Braconidae that larger parasitoids are issued from larger host larvae and larger females lay higher number of eggs [34]. In spite of the uniform size of *M. vitrata* caterpillars used for parasitization, we did observe a variable number of eggs in *T. javanus* females, which has been demonstrated to depend on abiotic or biotic factors in the Agathidinae *B. vulgaris* and *A. gibbosa* [9, 11]. For instance, the number of eggs laid by *B. vulgaris* varies depending on the size of the wasp female and on temperature at adult emergence, whereasmating is known to decrease egg load in *A. gibbosa*. In three-day-old mated *T. javanus* females, the mean number of mature eggs was much smaller than the number of immature ones (50 and 357, resp.), suggesting a large potential fecundity.As generally observed inkoinobiont species including someAgathidinae [35], this couldbe related to the ability of *T. javanus* females to continue oogenesis after emergence, provided there are sufficient protein sources to maintain oogenesis and complete egg maturation. In fact, the impact of adult life time protein deficiency on oogenesis has been previously documented in *Microterys flavus* (Hymenoptera: Encyrtidae) [36]. Like for *A. pumila* [10], *T. javanus* females do not host-feed and may probably need to take additional protein from nonhost food sources after emergence, possibly slowing down egg maturation.

As described for other koinobiont species that attack hosts at early stages [35], *T. javanus* displays very tiny eggs (0.1mm in length and 0.025mm for its larger width). This minute size and the tear shape of *T. javanus* eggs correspond to previous descriptions of Agathidinae eggs such as those produced by *B. vulgaris* (Cress.), *A. pumila,* and *A. gibbosa* [9–11]. *T. javanus* females are synovigenic; that is, they emerge with high numbers of immature eggs and only few mature eggs but continue to produce eggs throughout the adult stage, implying that females can start to oviposit in host caterpillars just after emergence. This ability to start oviposition just after emergence has been mentioned in other Agathidinae species, that is, *A. gibbosa*, *A. pumila,* and *B. vulgaris* [9–11]. In contrast to what was observed in the ovarioles of *A. pumila* and most Braconidae, however, both developing and mature eggs were found at the same level in the basal part of *T. javanus* ovariole. This suggests that *T. javanus* females might have developed amechanismallowing themto lay only mature eggs in the host.

The current mass-rearing protocol, using three-day-old *T. javanus* females, stems from a desire to maximize the production of mated females as recommended for parasitoid rearing in biological control programs [37, 38]. Our findings, however, show that egg production was relatively low (40.8 ± 8.6) during the first three days following adult emergence, suggesting that better outputs could be obtained using females older than three days. Our results also show that egg production in *T. javanus* is influenced by the size or instar of the caterpillar host. From a mass-rearing perspective, this suggests that the overall fecundity of *T. javanus* could be improved by selecting second instar caterpillars. However, under field conditions, the fecundity of foraging *T. javanus* females could be influenced by the size of the available *M. vitrata* life stages. Also, along with preliminary observations that *T. javanus* females did not perform host feeding on *M. vitrata* caterpillars, it will be important to investigate how feeding on sugar sources may impact the fecundity of *T. javanus* females. In fact, synovigenic parasitoids that do not feed on host are usually able to use sugar foods for oogenesis [18]. Notably, cowpea itself, the major crop hosting caterpillars of *M. vitrata*, secretes extrafloral nectar [39], which may provide an adequate source of sugar food for foraging female parasitoids.We expect similar extrafloral nectar to be present on other important, wild-occurring host plants such as *Sesbania rostrata* (Fabales: Fabaceae) and *Tephrosia platycarpa* (Fabales: Fabaceae), known to harbor important pod borer populations [40], which might also be visited by foraging *T. javanus* females.

## 5 Conclusion

Biological, rather than pesticidal, control of *M. vitrata* offers numerous advantages, especially in poor rural areas where the cost of pesticides, along with human and environmental exposures, becomes unsustainable or prohibitive. Even with pesticides, however, difficulty in recognizing the presence of *M. vitrata* prior to destructive crop predation makes conventional crop protectionmethods challenging. Our findings provide the first baseline information toward elucidating several factors influencing the reproductive biology in *T. javanus*, a promising biological control candidate against *M. vitrata* in West Africa. The fact that *T. javanus*, along with its high level of potential fecundity, may also demonstrate a greater facility for identifying and taking advantage of the presence of *M. vitrata* potentially enhances its use as a biological control to a great degree, while also affording the many cost, human, and environmental advantages of not using chemical pesticides.

## Conflicts of Interest

All authors declare no conflicts of interest.
